# Supplementary material for: Pre-existing antibody levels negatively correlate with antibody titers after a single dose of BBV152 vaccination
Source: Nat Commun. 2022 Jun 15;13:3451. doi: 10.1038/s41467-022-31170-1 (PMC9199457; doi:10.1038/s41467-022-31170-1)
Supplement: Supplementary file 1 — Supplementary Information [file 41467_2022_31170_MOESM1_ESM.pdf]

Supplementary Figure S1

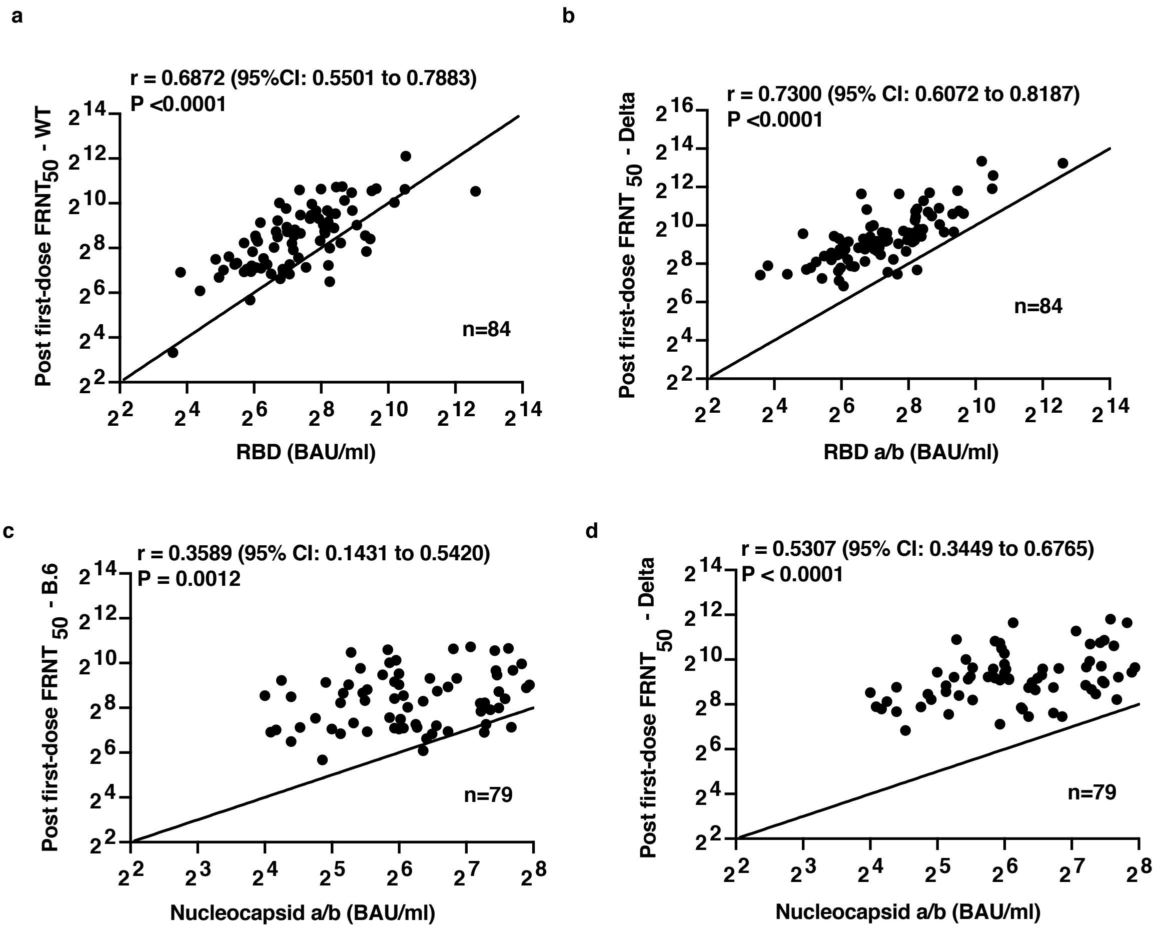

Supplementary Figure S1: Neutralization antibody titers by FRNT correlate with RBD and N antibody titers by ELISA. Spearman correlation (r) between RBD-ELISA binding antibody units (BAU/ml) and post-vaccination $FRNT_{50}$ titers against the (a) B.6 lineage (WT) or the (b) Delta variant virus. Spearman correlation (r) between N-ELISA binding antibody units (BAU/ml) and post-vaccination $FRNT_{50}$ titers against the (c) B.6 lineage (WT) or the (d) Delta variant virus. Two-tailed t test P values are indicated.

Supplementary Figure S2

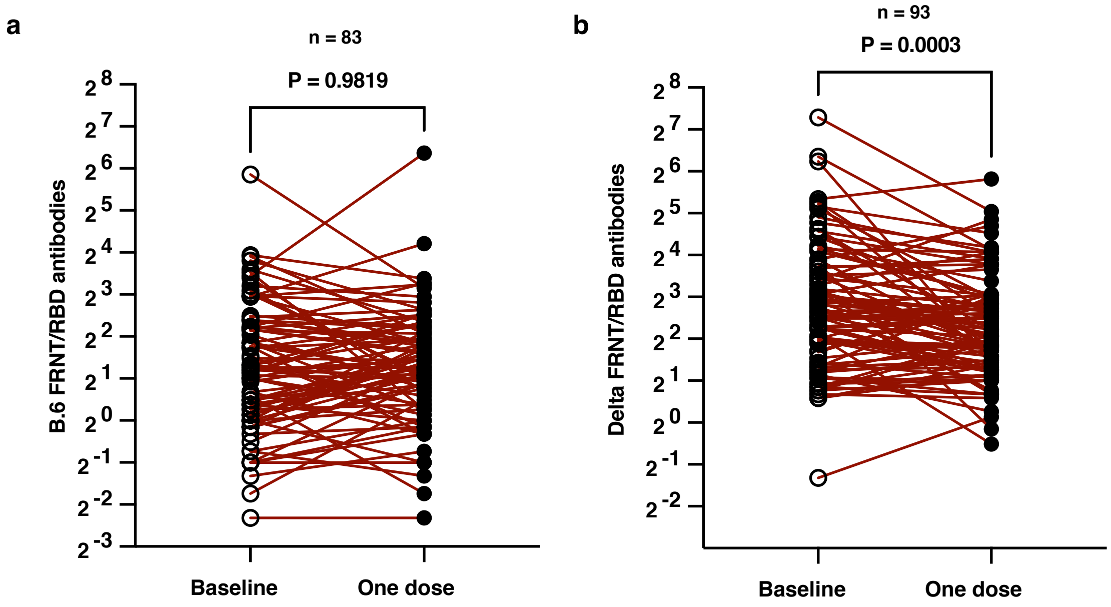

Supplementary Figure S2: Ratio of Neutralizing antibody titers to RBD after single dose of BBV152 vaccination. (a) The ratio of GMT of FRNT50 of B.6 lineage (n =83 independent values) or (b) the Delta variant to RBD ELISA was calculated in baseline and post-one dose vaccination with BBV152 (n=93 independent samples). Two-tailed P values were estimated by Wilcoxon matched-pairs signed rank test.

SUPPLEMENTARY TABLE 1

REAGENTS OR RESOURCES	SOURCE	IDENTIFIER
**Antibodies**	Immunostaining	
SARS-CoV-2 / 2019-nCoV Spike / RBD Antibody, Rabbit PAb	Sino Biological	Cat. No. 40592-T62<br>Lot No. HD15SE2803
SARS CoV-2 Nucleocapsid Antibody (4h2), Mab, mouse	GenScript	Cat. No. A02048-1<br>Lot No. H2011012
Goat Anti-Rabbit IgG (H+L) Horseradish Peroxidase conjugate	Invitrogen	Cat. No. G-21234<br>Lot No. 2321833
Goat anti-Mouse IgG (H+L) Cross-Adsorbed Secondary Antibody, HRP	Invitrogen	Cat. No. A16072<br>Lot No. 66-113-110220
HRP-labelled Goat Anti- Human IgG Fcγ specific	Jackson ImmunoResearch	Cat. No. 109-035-170<br>Lot No. 148542
**Plasmids for recombinant protein expression**		
Nucleocapsid protein of SARS-CoV-2	In-house purified	BEI resources: NR-53507
Receptor Binding domain (RBD) of SARS-CoV-2 Spike protein	In-house purified	BEI resources: NR-52422
**Virus strains**		
SARS-CoV-2 - B.6 lineage	Genbank accession: MW422884.1	
SARS-CoV-2 - B.1.1.617.2 (Delta variant)	Genbank accession: MZ356566.1	
SARS-CoV-2 - B.1.1.529 (Omicron variant - BA.1 sub-lineage)	GISAID-accession: EPI_ISL_6716890	
**Media and chemicals**		
Dulbecco's Modified Eagle Medium (DMEM), High glucose	HiMedia	Cat. No. AL007A
Expi293™ Expression Medium	Thermo Fisher/Gibco	Cat. No. A1435101
Expifectamine™ 293 Transfection kit	Thermo Fisher/Gibco	Cat. No. A14524
Penicillin-Streptomycin Antibiotic solution 100X liquid (PS)	HiMedia	Cat. No. A001
Minimum Essential Media (MEM)	Thermo Fisher/Gibco	Cat. No.11090073
Penicillin-Streptomycin-Glutamine (PSG) 100 X	Thermo Fisher/Gibco	Cat. No.10378016
Non-Essential Amino Acids Solution (100 X)	Thermo Fisher/Gibco	Cat. No.1140050

Fetal Bovine Serum (FBS)	Thermo Fisher/Gibco	Cat. No. 16140-071
Trypsin-EDTA (0.25%), phenol red	Thermo Fisher/Gibco	Cat. No. 25200072
1 M HEPES Buffer solution	Thermo Fisher/Gibco	Cat. No. 15630056
Sodium Bicarbonate Solution (7.5%)	Thermo Fisher/Gibco	Cat. No: 25080094
Carboxymethylcellulose (CMC) medium viscosity	Sigma	Cat. No. C4888
TrueBlue™ Peroxidase Substrate	Sera Care KPL	Cat. No: 5510-0050
1x Dulbecco's Phosphate Buffered Saline (DPBS)	Thermo Fisher/Gibco	Cat. No. 21600010
Hydrogen peroxide solution 30% w/v	Merck	Cat No. 216763
Formalin 37%	Merck	Cat. No. 1.94989.0521
Tween-20	Sigma	Cat. No. P-1379
Skimmed milk powder	Bio-Rad	Cat. No. 1706404
3,3’, 5,5’ tetramethylbenzidine (TMB)	BD Biosciences	Cat. No. 555214
Triton X-100	Sigma	Cat. No. T8787
Sodium Chloride (NaCl)	HiMedia	Cat. No. MB023-500 G
Potassium Chloride (KCl)	HiMedia	Cat. No. MB043-500 G
Sodium hydrogen phosphate (Na2HPO4)	HiMedia	Cat No. MB126-500 G
Potassium dihydrogen phosphate (KH2PO4)	Merck	Cat. No. 1.93605.0521
Vacutainer	BD Biosciences	Cat. No. 367954
**Cell lines**		
Vero E6	ECACC	Cat. No. 85020206
Calu-3 cells	ATCC	ATCC-HTB-55
Expi293F™ cells	Thermo Fisher/Gibco	Cat. No. A14527
**Software and Algorithms**		
Prism (9.3.1)	GraphPad	
SoftMax pro GXP 7.1	Molecular Devices	
AID EliSpot 8.0 iSpot software	AID	
Gen5 (3.10)	BioTek	
Microsoft Excel (v 16.16.27)	Microsoft (2016)	
Microsoft Word (v 16.16.27)	Microsoft (2016)	
