# Supplementary material for: Pre-existing antibody levels negatively correlate with antibody titers after a single dose of BBV152 vaccination
Source: Nat Commun. 2022 Jun 15;13:3451. doi: 10.1038/s41467-022-31170-1 (PMC9199457; doi:10.1038/s41467-022-31170-1)
Supplement: Supplementary file 3 — Reporting Summary [file 41467_2022_31170_MOESM3_ESM.pdf]

nature portfolio

Corresponding author(s): Guruprasad R. Medigeshi

Last updated by author(s): 2022/05/17

# Reporting Summary

Nature Portfolio wishes to improve the reproducibility of the work that we publish. This form provides structure for consistency and transparency in reporting. For further information on Nature Portfolio policies, see our Editorial Policies and the Editorial Policy Checklist.

## Statistics

For all statistical analyses, confirm that the following items are present in the figure legend, table legend, main text, or Methods section.

| n/a | Confirmed | |
|---|---|---|
| ☐ | ☒ | The exact sample size (*n*) for each experimental group/condition, given as a discrete number and unit of measurement |
| ☐ | ☒ | A statement on whether measurements were taken from distinct samples or whether the same sample was measured repeatedly |
| ☐ | ☒ | The statistical test(s) used AND whether they are one- or two-sided<br>*Only common tests should be described solely by name; describe more complex techniques in the Methods section.* |
| ☐ | ☒ | A description of all covariates tested |
| ☐ | ☒ | A description of any assumptions or corrections, such as tests of normality and adjustment for multiple comparisons |
| ☐ | ☒ | A full description of the statistical parameters including central tendency (e.g. means) or other basic estimates (e.g. regression coefficient) AND variation (e.g. standard deviation) or associated estimates of uncertainty (e.g. confidence intervals) |
| ☐ | ☒ | For null hypothesis testing, the test statistic (e.g. *F*, *t*, *r*) with confidence intervals, effect sizes, degrees of freedom and *P* value noted<br>*Give P values as exact values whenever suitable.* |
| ☒ | ☐ | For Bayesian analysis, information on the choice of priors and Markov chain Monte Carlo settings |
| ☒ | ☐ | For hierarchical and complex designs, identification of the appropriate level for tests and full reporting of outcomes |
| ☒ | ☐ | Estimates of effect sizes (e.g. Cohen's *d*, Pearson's *r*), indicating how they were calculated |

*Our web collection on statistics for biologists contains articles on many of the points above.*

## Software and code

Policy information about availability of computer code

| | |
|---|---|
| Data collection | ELISA data were collected using Gen5 (v 3.10) software from BioTek. All the data were entered in Microsoft Excel v 16.16.27. |
| Data analysis | Data were analysed and final graphs were prepared using GraphPad Prism (Version 9) software. For comparison analysis, Stata ver 11.2 (StataCorp LP College Station, TX) was used. FRNT data was calculated by SoftMax Pro GxP software v7.7.1 (Molecular Devices). Microsoft Word (v 16.16.27) was used for preparing the manuscript. |

For manuscripts utilizing custom algorithms or software that are central to the research but not yet described in published literature, software must be made available to editors and reviewers. We strongly encourage code deposition in a community repository (e.g. GitHub). See the Nature Portfolio guidelines for submitting code & software for further information.

## Data

Policy information about availability of data

All manuscripts must include a data availability statement. This statement should provide the following information, where applicable:

- Accession codes, unique identifiers, or web links for publicly available datasets
- A description of any restrictions on data availability
- For clinical datasets or third party data, please ensure that the statement adheres to our policy

All data generated or analyzed during this study are included in this manuscript. The virus strains used in this study have the following accession numbers: SARS-CoV-2 - B.6 lineage (Genbank accession: MZ356566.1), SARS-CoV-2 - B.1.1.617.2 (MZ356566.1) and SARS-CoV-2 - B.1.1.529 (BA.1 sub-lineage) (GISAID-accession:EPI_ISL_6716890). Source data is provided with this paper.

# Field-specific reporting

Please select the one below that is the best fit for your research. If you are not sure, read the appropriate sections before making your selection.

☒ Life sciences ☐ Behavioural & social sciences ☐ Ecological, evolutionary & environmental sciences

For a reference copy of the document with all sections, see nature.com/documents/nr-reporting-summary-flat.pdf

# Life sciences study design

All studies must disclose on these points even when the disclosure is negative.

| | |
|---|---|
| Sample size | No sample size calculation was performed as this was an exploratory study intending to measure antibody responses in participants after a single dose of vaccination. The study was initiated after the end of second wave of COVID-19 in India which resulted in around 17 million reported cases of COVID-19 and with only around 10% of the population vaccinated. There were no studies on how an inactivated vaccine (COVAXIN) would elicit the antibody response in participants with or without prior exposure to SARS-CoV-2 infection. This information was needed to inform vaccination policy on booster doses and to prioritize high risk groups. We planned to collect samples from 100 participants based on the logistical capabilities. We were able to enrol 94 participants who provided both the baseline and follow-up blood sample for the study. |
| Data exclusions | No data were excluded |
| Replication | All the assays involved testing the independent clinical samples (n=94) for N, RBD ELISA and neutralization assays with multiple variants. All the assays were performed once with two technical replicates and with appropriate secondary reference controls which were calibrated against the WHO reference standard. We are part of the centralized network laboratories of CEPI and all the assays are either verified within the network or have been accredited under the ISO 17025:2017 standard. |
| Randomization | This is not a clinical trial, therefore, randomization is not applicable. |
| Blinding | This is not a clinical trial. Therefore, blinding is not applicable. |

# Reporting for specific materials, systems and methods

We require information from authors about some types of materials, experimental systems and methods used in many studies. Here, indicate whether each material, system or method listed is relevant to your study. If you are not sure if a list item applies to your research, read the appropriate section before selecting a response.

## Materials & experimental systems

| n/a | Involved in the study | |
|---|---|---|
| ☐ | ☒ | Antibodies |
| ☐ | ☒ | Eukaryotic cell lines |
| ☒ | ☐ | Palaeontology and archaeology |
| ☒ | ☐ | Animals and other organisms |
| ☐ | ☒ | Human research participants |
| ☒ | ☐ | Clinical data |
| ☒ | ☐ | Dual use research of concern |

## Methods

| n/a | Involved in the study | |
|---|---|---|
| ☒ | ☐ | ChIP-seq |
| ☒ | ☐ | Flow cytometry |
| ☒ | ☐ | MRI-based neuroimaging |

## Antibodies

| | |
|---|---|
| Antibodies used | Anti-spike rabbit polyclonal antibody (Sino Biologicals, Beijing, China; 40592-T62; Lot no. HD15SE2803); SARS CoV-2 Nucleocapsid Antibody (4h2), Mab, mouse (GenScript, Cat. No. A02048-1; Lot no. H2011012); HRP-conjugated Goat anti-rabbit antibody (Invitrogen, Cat. No. G-21234; Lot no. 2321833); Goat anti-Mouse IgG (H+L) Cross-Adsorbed Secondary Antibody, HRP (Invitrogen, Cat. No. A16072; Lot no. 66-113-110220); Anti-human-IgG-Fcgamma specific HRP-conjugated secondary antibody (Jackson Immunoresearch, Cat. no. 109-035-170; Lot no. 148542). |
| Validation | The specific IgG was purified by SARS-CoV-2 / 2019-nCoV Spike/RBD affinity chromatography for the anti-spike rabbit polyclonal antibody. The antibody has cross-reactivity with SARS-CoV Spike S1 and SARS-CoV Spike RBD as stated in the data sheet. The antibody has been validated by the manufacturer for western blot and ELISA. IHC, FCM, IF, IP et al. applications haven't been validated.<br>The FRNT method using anti-spike antibody and anti-rabbit secondary antibody has been reported earlier (https://doi.org/10.1038/s41596-021-00536-y).<br>SARS CoV-2 Nucleocapsid Antibody (4h2), Mab has been purified using Protein-A affinity column and has been validated for ELISA and Western blotting as stated in the data sheet provided by the manufacturer. The antibody is specific to SARS-CoV-2 N protein by ELISA.<br>The HRP-conjugated Goat anti-rabbit antibody is cross-adsorbed against human IgG, human serum, mouse IgG, mouse serum and bovine serum and is specific to rabbit IgG. The antibody has been validated in WB, IHC, IP and ELISA. |

The goat anti-mouse IgG (H+L) Cross-Adsorbed Secondary Antibody, HRP (Cat. No. A16072) has been validated in WB, IHC and ELISA. The antibody has been cross-adsorbed against bovine, horse, human, pig and rabbit serum proteins and affinity purified as per the datasheet provided by the manufacturer.
For anti-human-IgG secondary antibody, the specification sheet of Jackson Immuno research states as follows "Based on immunoelectrophoresis and/or ELISA, the antibody reacts with the Fc portion of human IgG heavy chain but not with the Fab portion of human IgG. No antibody was detected against human IgM or IgA, or against non-immunoglobulin serum proteins. The antibody has been tested by ELISA and/or solid-phase adsorbed to ensure minimal cross-reaction with bovine, mouse, and rabbit serum proteins, but it may cross-react with immunoglobulins from other species".

## Eukaryotic cell lines

Policy information about cell lines

| | |
|---|---|
| Cell line source(s) | VeroE6 (European collection of authenticated cell cultures, Cat.no. 85020206); Calu-3 (American Type Culture Collection, ATCC-HTB-55); Expi293F™ cells (ThermoFisher, Cat. No. A14527) |
| Authentication | Cell lines were procured from the above sources directly and have not been authenticated |
| Mycoplasma contamination | All cell lines are routinely tested for mycoplasma using a commercial PCR kit (Southern Biotech Cat. No. 13100-01) and were found to be negative for mycoplasma. |
| Commonly misidentified lines (See ICLAC register) | None |

## Human research participants

Policy information about studies involving human research participants

| | |
|---|---|
| Population characteristics | A total of 94 (40 females) subjects were enrolled. Median age of the subjects was 30.5 yrs (range: 18-67 yrs). |
| Recruitment | All adults of > 18 years of age who came for the first dose of vaccine were eligible to participate in this study including those who had recovered from the COVID-19 in the recent past. |
| Ethics oversight | The study was approved by the Institutional ethics committees for human research at ESIC Hospital and Medical College and THSTI. |

Note that full information on the approval of the study protocol must also be provided in the manuscript.
